# IL-10: A Multifunctional Cytokine in Viral Infections

**DOI:** 10.1155/2017/6104054

**Published:** 2017-02-20

**Authors:** José M. Rojas, Miguel Avia, Verónica Martín, Noemí Sevilla

**Affiliations:** Centro de Investigación en Sanidad Animal (CISA), Instituto Nacional de Investigación y Tecnología Agraria y Alimentaria (INIA), Valdeolmos, Madrid, Spain

## Abstract

The anti-inflammatory master regulator IL-10 is critical to protect the host from tissue damage during acute phases of immune responses. This regulatory mechanism, central to T cell homeostasis, can be hijacked by viruses to evade immunity. IL-10 can be produced by virtually all immune cells, and it can also modulate the function of these cells. Understanding the effects of this multifunctional cytokine is therefore a complex task. In the present review we discuss the factors driving IL-10 production and the cellular sources of the cytokine during antiviral immune responses. We particularly focus on the IL-10 regulatory mechanisms that impact antiviral immune responses and how viruses can use this central regulatory pathway to evade immunity and establish chronic/latent infections.

## 1. IL-10 and the Complex Interplay between Its Cellular Sources and Targets

Antiviral immune responses ideally eliminate replicating virus and viral reservoirs without host damage. However, in many infections, severe complications could occur due to excessive immune activation. To prevent host tissue damage, immunoregulatory cytokines control the magnitude of these immune responses. IL-10 is a key component of this cytokine system that regulates and suppresses the expression of proinflammatory cytokines during the recovery phases of infections and consequently reduces the damage caused by inflammatory cytokines [1, 2]. IL-10 binds IL-10R, a dimeric receptor composed of a high affinity IL-10R1 chain predominantly expressed on leukocytes and unique to IL-10 recognition, and an ubiquitously expressed IL-10R2 chain involved in the recognition of other cytokines from the IL-10 family (IL-22, IL-26, IL-28A, IL-28B, and IL-29) [3, 4]. The interaction of IL-10 with IL-10R triggers the Jak-STAT signaling pathway, leading to STAT1, STAT3, and, in some instances, STAT5 activation. STAT3 is critical for IL-10 effects on immune cells [5–7].

As its specific receptor (IL-10R1) expression indicates, IL-10's broad spectrum of cellular targets includes virtually all leukocytes. IL-10 is considered a master negative regulator of inflammation. Blockade in the IL-10 pathway typically results in prolonged and exaggerated immune responses to antigens that can lead to immunopathology. Initially identified as a Th1 inhibitory factor secreted by Th2 cells [8], IL-10 is now known to be produced by a variety of innate and adaptive immune cells, including macrophages, dendritic cells (DCs), natural killer (NK) cells, CD4, CD8, *γδ* T cells, and B cells (reviewed in [4, 9, 10]). Untangling the complex interplay between IL-10 sources and target cells during immune responses remains an outstanding challenge. For instance, systemic administration of IL-10 for autoimmune therapy proved to be paradoxically proinflammatory [11, 12], whereas localized IL-10 delivery usually proves to be therapeutic [13–15]. Spatial delivery of IL-10 signaling is therefore crucial to its effects.

Autoimmune disease models in IL-10-deficient mice have helped elucidate the role of this cytokine in T cell homeostasis in the periphery. They also highlight the complex link between IL-10's source and its role. IL-10-deficient mice develop spontaneous enterocolitis typically driven by microbial insult and dependent on T cell responses [16–18]. When these mice are bred in pathogen-free environments or when MyD88 (a key component for pathogen recognition receptor (PRR) signaling) is also knocked out, colitis does not occur implicating the gut microflora as a causal agent [16–20]. IL-10 thus maintains T cell tolerance to commensal microflora in the gut. Treg cells are critical in the prevention of spontaneous colitis in this model [21, 22]. When IL-10 deficiency is restricted to the Treg cell compartment, mice develop colitis [22]. Although Treg cells are the source of IL-10 that maintains peripheral tolerance, they also need to sense IL-10 to provide protection, as IL-10R-deficient Treg cells cannot impair disease development [23]. Restricting IL-10 deficiency to myeloid cells does not cause colitis which confirms that macrophages are not the main source of protective IL-10 in this model [24]. IL-10 produced by macrophages could however partly contribute to colitis protection, as it triggers Treg cell protection when anticommensal T cells are adoptively transferred into a sensitive host [25]. Importantly, deficiency in IL-10R signaling in macrophages leads to colitis development [24, 26]. IL-10 signaling appears necessary for macrophages to trigger their anti-inflammatory functions. Macrophages thus act as intermediates in the maintenance of tolerance. IL-10 produced during the initial inflammation in the gut probably drives IL-10 production by Treg cells, which in turn limits macrophage-induced activation of anticommensal T cells, maintains peripheral T cell tolerance, and controls immunopathology.

This well-studied autoimmune model shows how IL-10 produced locally acts as a natural negative feedback mechanism that controls inflammation and maintains immune homeostasis in the periphery. Indeed, IL-10 deficiency aggravates several experimental autoimmune disorders [27–29], illustrating the central role of this cytokine in immune regulation.

IL-10 is also important in controlling viral immunity. Studies using lymphocytic choriomeningitis virus (LCMV) infections with strains that provoke either acute or persistent infections have helped understand the role of IL-10 in viral infections. IL-10 acts as an immunoregulator, inhibiting proinflammatory responses from innate and adaptive immunity and preventing tissue damage due to exacerbated adaptive immune response. However, viruses have evolved mechanisms that exploit the immunoregulatory function of IL-10 for immune evasion, suppression, and tolerance, promoting their own survival. As a result, viruses can persist for life in infected hosts possessing otherwise competent immune responses. The effects of pleiotropic IL-10 during the course of infection are nonetheless multiple and the subtle IL-10-governed mechanisms that balance inflammation and immunoregulation are still subject to plenty of attention. In this review, we will discuss the role of IL-10 in immune cells during acute infections and the IL-10-dependent mechanisms that viruses use to drive viral persistence.

## 2. IL-10 in Acute Viral Infection

### 2.1. Early IL-10 Induction and Effects on Innate Immunity

**Figure 1:**
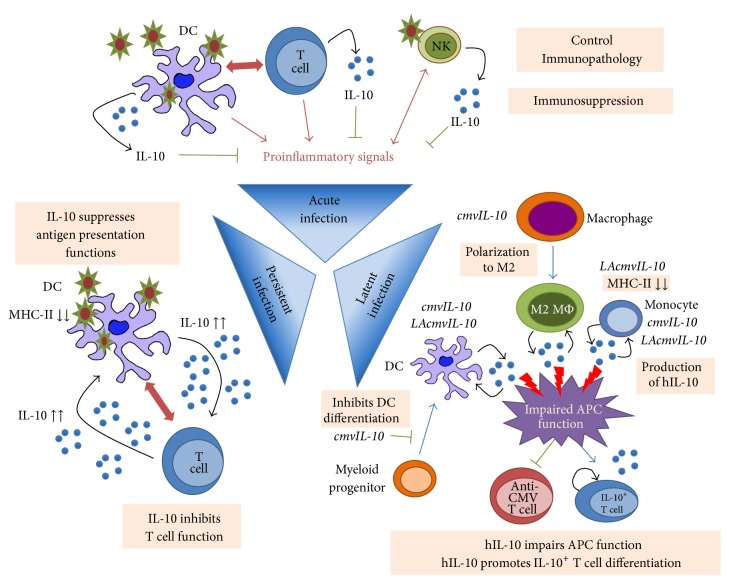
IL-10 role in viral infections. During acute infections, proinflammatory signals are produced by DCs after recognition of pathogen patterns. In parallel, NK cells recognizing pathogen patterns and/or stimulated by proinflammatory signals further enhance inflammation. In this proinflammatory context, DC can promote antiviral T cell responses that clear the infection. Activation of DC, T cells, and NK cells also results in the production of the immunoregulatory cytokine IL-10 to balance inflammation. In this context, IL-10 expression controls immunopathology and leads to the resolution of the inflammation and T cell responses once the pathogen is cleared. During persistent infections, the virus exploits the production of IL-10 by DCs to exhaust antiviral T cells. High IL-10 levels produced by DCs suppress their antigen presenting capacity and lead to inefficient T cell activation. Chronic antigen presence further exhausts T cells and induces IL-10 production. T cells therefore become “tolerant” to viral antigens and infection persists. To establish chronicity and latent infections, the virus produces viral IL-10 homologs that favor anti-inflammatory responses. In human cytomegalovirus infection, cytomegalovirus-encoded IL-10 (cmvIL-10) and latency-associated cytomegalovirus-encoded IL-10 (LAcmvIL-10) are produced in myeloid cells and impair their function. cmvIL-10 induces hIL-10 production in DCs, macrophages, and monocytes, impairs DC differentiation, and promotes M2 polarization of macrophages. LAcmvIL-10 also promotes hIL-10 production in DCs and monocytes and impairs monocyte presenting capacity. IL-10 viral homologs induce human IL-10 (hIL-10) production in myeloid cells that contributes to impairment of their antigen presenting cell (APC) function. This in turn probably limits anti-CMV T cells responses and promotes IL-10^+^ T cell development. Impaired APC function permits chronic infections, while IL-10^+^ T cells allow latent infections to persist.

During the early phase of infections, viruses typically trigger PRR engagement after pathogens-associated molecular patterns (PAMPs) or danger-associated molecular patterns (DAMPs) recognition (reviewed in [30]). PAMP and DAMP recognition drives the antiviral state in antigen-presenting cells (APC) and type I IFN production that initiate the innate immune response. Concomitant to the proinflammatory first line of defense triggered by PRR signaling, the immunoregulatory cytokine IL-10 is induced in DCs and macrophages (Figure 1) [31–37]. The regulation of IL-10 production in APC is complex and depends on cell type [37] and the integration of secondary activation signals such as type I IFN [34, 38], PGE_2_ [39], or CD40 ligation [40] that synergize with PRR signals. Moreover, IL-10 production in APC can be antagonized by the presence of IFN-*γ* [34, 41]. In macrophages, IL-10 production can be maintained through an autocrine IFN-*β* feedback loop [36]. In DC, IL-10 production depends on subtype-specific preprogrammed cytokine patterns [37, 40]. Kinetic studies indicate that IL-10 could be produced in late activation phase in APCs [33, 34], which suggests that IL-10 balances the proinflammatory signals induced by viral PAMPs. Early IL-10 production by APCs probably limits excessive inflammation and thus potential tissue damage.

NK and NKT cells are an essential effector arm of innate immunity that participates in the control of viral infections [42–45]. IL-10 has been shown to promote NK cell proliferation, cytokine production, and cytotoxicity in vitro [46–50], although in some in vivo settings it could modulate NK cell activity [51, 52]. IL-10 acts as a prosurvival factor in activated NK cells by inhibiting activation-induced cell death [53]. The cytokine thus appears to promote activated NK cell effector function. Interestingly, NK cells are also a source of IL-10 upon synergistic activation with IL-2 and IL-12 (Figure 1) [54–57]. IL-10-producing NK cells can control liver inflammation in acute murine cytomegalovirus (MCMV) infection [58] and therefore limit immunopathology in some organs. IL-10-producing NK cells could serve as an early control for excessive inflammation during the initiation of the immune response [59, 60], while their viremia-controlling effector functions are maintained. IL-10 produced in the early phase of antiviral innate immunity by APCs and NK cells is probably a counterbalance to proinflammatory signals that protect from tissue damage. Although in most cases IL-10 derived from innate immune cells is unlikely to affect the development of antiviral immunity, this source of IL-10 can be induced by some viruses to evade immunity, as described later.

### 2.2. IL-10 and Antiviral Cellular Responses

To eliminate intracellular pathogens like viruses the immune system typically uses cytotoxic CD8^+^ T lymphocytes (CTL), whose functions are armed by Th1 cells. CD8^+^ T cells are critical in antiviral immunity, since they can kill infected cells through the recognition of viral peptides presented on MHC I molecules. Th1 cells also recognize viral peptides presented by APC on MHC-II molecules. Th1 cells provide the “license to kill” to the virus-specific CD8^+^ T cells to differentiate into effector CTLs using professional APC as intermediates [61, 62]. This central mechanism of antiviral immunity can be modulated by IL-10 at different levels. High IL-10 levels act as a regulatory trigger that initiate the resolution of the acute phase of infection in which antiviral T cell populations contract [63].

#### 2.2.1. IL-10 Production by Antiviral T Cells

Currently it is well established that virtually all T cell subsets can produce IL-10 (reviewed in [64, 65]). IL-10 production appears thus to be embedded in the activation program of T cells. Indeed, at the height of the inflammatory response and once cellular immune responses are mounted, antiviral CD4^+^ and CD8^+^ T cells become the main sources of IL-10 (Figure 1) [66–72]. Th1 cells can produce IL-10 [73] in response to intracellular protozoan [74, 75], LCMV [72, 76], MCMV [77–79], or influenza [68] infections among others. IL-10 production in Th1 cells is driven by TCR engagement but is not directly regulated by T-bet, the master transcription regulator of Th1 cell programming [80, 81]. IL-27 (a proinflammatory cytokine belonging to the IL-12 family) is a potent inducer of IL-10 in Th cells [82–85]. Type I IFN can also induce IL-10 expression in CD4^+^ T cells [86, 87]. IL-10 production in Th cells therefore depends on secondary environmental signals upstream of STATs (such as IL-10 itself [5–7] and proinflammatory cytokines [88]) or SMADs (such as TGF-*β* [89]). It should be noted that chronic antigen stimulation results in IL-10-producing Th1 cells [72, 90, 91] unable to respond to pathogens. This natural regulatory mechanism that maintains T cell homeostasis in the periphery can be used to establish chronic infection as discussed later.

Effector CD8^+^ T cells can produce IL-10 during the acute phase of influenza virus [67, 68], respiratory syncytial virus [70], coronavirus infection [69], paramyxovirus simian virus 5 [71], or vaccinia [66] infections. The transcription factor BLIMP-1 is essential for IL-10 production in effector and memory CD8^+^ T cells [92]. BLIMP-1 is induced in CD8^+^ T cells through T cell help and can be sustained by proinflammatory signals (IL-27), T cell growth factors (IL-2) [92], and antiviral signaling like type I IFN [67]. It thus appears that antiviral and inflammatory signals elicited during viral infections trigger activated T cells to produce IL-10 as a feedback regulatory mechanism that limits excessive inflammation.

#### 2.2.2. IL-10 Uses APC as Intermediate to Modulate T Cell Responses

Although T cells become the main IL-10 producers during the acute phase of infection, IL-10 effects on T cell function are usually mediated through paracrine activity on DCs and macrophages (reviewed in [1, 9]). IL-10 recognition by APC skews their response towards a noninflammatory protissue repair phenotype [93–99]. IL-10 is a major regulator of the potent APC-derived inflammatory cytokine IL-12 [100] and promotes expression of its own mRNA in a positive feedback loop [101]. Exposure to IL-10 also leads to downregulation of costimulatory and MHC molecules on APCs [4, 102, 103] which limits the amount of antigen exposure T cells can receive. IL-10 also restricts the production of proinflammatory cytokines and chemokines that permit APC trafficking to the lymph nodes, thereby interrupting Th1 differentiation of naïve T cells [103, 104]. These elevated IL-10 levels impair de novo Th1 stimulation [105, 106] and trigger the resolution of the acute phase of infection in which antiviral T cell populations contract [63]. IL-10 therefore acts as a switch on APC that controls inflammation and ultimately interrupts T cell responses once pathogens are cleared.

#### 2.2.3. IL-10 Effects on Antiviral T Cells

Through its effects on APC, IL-10 can alter antiviral T cell function, although its effects on Th1 cells and CTLs are very different. Acute and chronic LCMV infection models have been essential to comprehend IL-10's crucial role in controlling antiviral T cell responses. IL-10 limits cytokine production and proliferation in antiviral Th1 cells [2, 104, 107]. When IL-10 regulatory action is removed (through IL-10R blockade or IL-10 deficiency), antiviral Th1 responses can prevent chronic LCMV infection [2, 31, 104, 107, 108]. IL-10 blockade increases the amount of Th1 cells in germinal centers [104], promotes Th1 priming [106], and enhances Th1 effector function and memory development [104, 107]. IL-10 thus appears central to the regulation of antiviral Th1 cell responses. Removal of the IL-10 “brake” on Th responses can lead to immunopathology following viral infection as illustrated by the increased neurologic disease detected in IL-10-deficient mice during fatal alphavirus encephalomyelitis [109]. This general regulatory mechanism prevents host immunopathology and controls the amplitude of Th1 cell responses during acute viral infections. This mechanism can nonetheless be exploited by viruses to promote chronic and persistent infections as discussed later.

In contrast to Th1 cells, CD8^+^ T cell effector functions (e.g., cytokine production and cytotoxicity) can be enhanced by IL-10 addition in vitro [4]. IL-10 blockade prior to LCMV infection only results in a modest increase in LCMV-specific CD8^+^ T cells 8 days after infection [104, 107], which indicates that IL-10 does not greatly alter antiviral CD8^+^ T cell priming. Nonetheless, IL-10 blockade/deficiency facilitates virus clearance by CD8^+^ T cells in chronic LCMV infections [2, 31, 104, 107], which confirms that secondary CD8^+^ T cell responses are regulated by IL-10 [105]. It should be noted that the effects of IL-10 on CD8^+^ T cells could also depend on the strength of the antigenic signal, as CD8^+^ T cells recognizing different LCMV epitopes appear to have different IL-10 inhibition thresholds [104].

IL-10 has also been linked to CD8^+^ T cell memory differentiation [110, 111]. Recently IL-10 produced by Treg cells was shown to promote CD8^+^ T cell memory differentiation in LCMV infections by insulating a portion of CD8^+^ T cells from inflammatory signals during the resolution phase of the immune response [112]. Other reports have nonetheless indicated that IL-10 could impair CD8^+^ T cell memory development in the same infection [104], while others found no difference in the quality and quantity of CD8^+^ T cell memory development after IL-10 blockade [107]. These contradictory results obtained through different approaches (IL-10/IL-10R antibody blockade, IL-10-deficient mice, or adoptive transfer of IL-10-sufficient Treg cells) hint at a very delicately regulated system for CD8 memory development that could be controlled by T cell signal strength as well as spatial and temporal IL-10 delivery. This raises the intriguing possibility for a new facet in IL-10 biology whereby IL-10 dampening of CD8^+^ T cell responses could facilitate the differentiation of a portion of these cells into memory.

### 2.3. IL-10 and Antiviral Humoral Response

B cell-produced antibodies represent the other major arm of the adaptive immunity involved in virus clearance [113]. Most clinically effective vaccines not only require the induction of cellular immunity but also the production of neutralizing antibodies [114, 115]. Nonneutralizing antibodies can also participate in antiviral immunity as shown in LCMV infections where virus-specific nonneutralizing antibodies participate in virus clearance alongside CD4^+^ and CD8^+^ T cells [108]. The importance of B cell responses in viral immunity is also exemplified by the interference of viruses with humoral immunity. For instance, Bluetongue virus can affect antiviral antibody titers early in infection [116], and human immunodeficiency virus (HIV) can continuously mutate its antigenic determinants, a phenomenon known as antigenic drift, to evade neutralization by antibodies [117, 118].

Since IL-10 regulates B cell survival and differentiation [4], it could potentially control B cell responses to virus. IL-10 favors B cell effector function by stimulating plasma cell differentiation at the expense of B memory cells [4, 119]. Autocrine IL-10 production promotes B cell survival and Ig class switch [120–122]. In LCMV-infected mice, IL-10 however does not control B cell differentiation in the priming phase [104]. Moreover IL-10 blockade does not affect follicular Th cell numbers, a subpopulation of Th cells involved in B cell help and necessary for the generation of high affinity antibodies [104]. It thus appears that IL-10 may not directly affect B cell responses, although this has not been widely studied.

B cells could nonetheless be a source of IL-10 that could modify antiviral responses. IL-10 expression in B cells can be triggered by TLR engagement [123–125] and increases when B cells are activated in a context mimicking T cell and DC help, that is, through anti-Ig antibody, anti-CD40 antibody, and IL-12 [126]. Type I IFN that are typically produced during antiviral responses can also enhance TLR-induced IL-10 production in B cells [127, 128]. These reports indicate that IL-10 production is an integral part of B cell activation programming. However, the factors driving IL-10 production in B cells during immune responses are not fully understood.

A B regulatory cell population (Breg) has been described [129, 130] and can be a principal source of IL-10. No precise Breg cell markers have so far been defined (reviewed in [131, 132]), but these cells are potent inhibitors of autoimmune inflammation through their IL-10 production [133, 134]. Breg cells can suppress* Listeria monocytogenes* [135] or* Salmonella typhimurium* [136] clearance. These cells can therefore also modulate responses to infections. The observation that Breg cells can be therapeutic in allergy [137] indicates that IL-10 produced by Breg cells could have systemic activity. IL-10-producing Breg cell numbers increase in coxsackie virus-induced acute myocarditis model [138]. In MCMV murine infections, IL-10 expression in B cells can suppress MCMV-specific CD8^+^ T cells responses [139, 140]. Moreover, IL-10-producing Breg cells could promote chronic MCMV brain infection [141]. In HIV patients, IL-10-producing Breg cells are elevated in peripheral blood of untreated patients and can suppress virus-specific CD4^+^ and CD8^+^ T cell activity in vitro [142]. Similarly, in chronic hepatitis B virus (HBV) patients, IL-10-producing B cells are elevated in the periphery and suppress HBV-specific CD8^+^ T cell responses [143]. IL-10-producing B cells have therefore the capacity to create an immunoregulatory milieu unsuitable for cellular immunity. The localized effects of IL-10 on T cells however suggest that B cell-derived IL-10 would probably affect effector T cell activity in specific settings. Further work will be required to clarify both how B cell-derived IL-10 influences antiviral responses and how IL-10 modulates antiviral B cell responses.

### 2.4. IL-10 and Virus Clearance

Although IL-10 acts as an immune brake on inflammation, its overall effects on antiviral immune responses can be complex and depend on the virus, site of infection, timing of the antiviral immune response, and so forth. For instance, high IL-10 plasma levels could be protective in early responses to HIV but become detrimental during acute infection as they promote virus persistence [144].

In some settings, IL-10 expression can contribute to virus clearance. In influenza infections, coproduction of IL-10 and IFN-*γ* facilitates anti-influenza antibody accumulation in the lung mucosa [145]. Thus IL-10 not only limits immunopathology in this case but also supports adaptive immunity. In cutaneous vaccinia virus infections, IL-10-producing T cells have been linked to lesion control [66], which suggests that local IL-10 effects may be multiple and depend on the organ and microenvironment.

IL-10's supportive role for effective virus clearance is very apparent in CNS infections. Virus-induced encephalitis results from an excessive immune-induced inflammation designed to control viral infection. IL-10-deficiency aggravates this immunopathology in* Flavivirus* or* Coronavirus* infections with CNS tropism [63, 146–149]. In these CNS infections, IL-10 usually improves virus control, although this outcome probably results from direct and indirect effects of the cytokine. In CNS immune responses to the coronavirus mouse hepatitis virus, CD4^+^ T cells and CD8^+^ T cells are the initial sources of IL-10 [63]. Once the viral load is controlled, IL-10-producing CD8^+^ T cells diminish while IL-10-producing CD4^+^ T cells remain [63]. IL-10 produced during the immune response peak could enhance CD8 activity while limiting APC-driven inflammation. During this resolution phase, natural CD4^+^ CD25^+^ Treg cells are the main source of IL-10. However transition in the IL-10 source from natural Treg cells to T regulatory 1- (Tr1-) like CD4^+^ CD25^−^ cells could be a sign of CNS viral persistence [63] and indicate chronic antigen stimulation. In infection with the* Flavivirus* Japanese Encephalitis virus, IL-10-producing CD4^+^ Foxp3^+^ natural Treg cells improve survival in a murine model probably by controlling the immunopathology [148]. In other organs, modulation of immunopathology by IL-10 during infection is not solely reliant on Treg cell activity. In MCMV acute infection, NK cells are the main IL-10 source that modulates immunopathology in liver [58], while IL-10-producing Breg cells probably participate in neuroinflammation control [141]. IL-10 regulatory mechanisms are therefore essential to control severe inflammatory responses produced by viral infections and can thereby, albeit indirectly, be essential for virus clearance.

In an adequate acute immune response, IL-10 presence should not affect virus clearance; however sustained expression during immune priming or secondary responses can favor persistence or chronic infections. This fine balance between the inflammatory response crucial to virus clearance and the IL-10-mediated immune regulation necessary for T cell homeostasis and host tissue protection can be subverted by viruses to allow replication and spreading.

## 3. IL-10 in Chronic Viral Infections

Persistent or chronic viral infections are not cleared by the host immune response and result in long-term equilibrium between the host and the virus. Several factors can contribute to this persistence such as viral immune evasion mechanisms, impaired viral clearance facilitated by the host-regulated immunosuppression, or, as for herpesviruses, manipulation of the host immune environment to enable persistence (latency). We will next review different mechanisms used by viruses to induce chronicity or persistence, in which either host IL-10 is involved as a regulating cytokine or viruses have evolved mechanisms that mimic IL-10 function, such as IL-10 viral homologs.

### 3.1. Persistent Viral Infections

Persistent infections such as those established by hepatitis C virus (HCV), HBV, and HIV are of particular interest in human health due to their high rates of morbidity and mortality as well as the lack of efficient therapies. Impaired viral clearance can result from viral evasion of the immune response or be assisted by the host-regulated immunosuppression. More precisely, CD4^+^ T cells and CD8^+^ T cells lose their effector functions and are unable to control viral infections, a phenomenon called T cell exhaustion [150] (Figure 1). CD8^+^ T cells lose the ability to produce antiviral cytokines, to kill infected cells, and to proliferate in response to antigen stimulation [151]. Similarly, CD4^+^ T cells show impaired cytokine production and lack of proliferation [90]. This loss of T cell function has been described in persistent infections with HCV, HBV, HIV, and LCMV, suggesting that a conserved mechanism of immunosuppression may downregulate T cell function. These mechanisms produce gene expression changes in T cells, including inhibitory receptor induction [152, 153], production of soluble factors such as TGF-*β* [154], or elevated systemic IL-10 levels [2, 155, 156]. The programmed death-1 (PD-1)/PD-ligand(L)1 inhibitory pathway actively suppresses T cell responses and can also participate in the establishment of persistent infections [106, 152]. Although PD-1 contributes to T cell exhaustion, a common characteristic of these persistent infections is elevated IL-10. This has been described for HCV and HIV infections in which high IL-10 levels in the early/acute phase are associated with progression to persistence [157–160], which suggests that this is an evolutionarily conserved mechanism in persistent viral infections with clinical relevance.

Studies on LCMV persistent infection have helped elucidate the mechanisms by which IL-10 can mediate persistent infections. Infection of adult mice with Armstrong (Arm) LCMV strain results in acute infections that are efficiently cleared within 7–10 days by anti-LCMV CD8^+^ CTLs. By contrast, the LCMV clone 13 (Cl13) induces a persistent infection that suppresses cellular and humoral responses. Cl13 infection of DCs results in cell loss in this compartment during the first week of infection and plays a relevant role in establishing persistence [161–163]. Among the different host factors that play a role in immunosuppression in Cl13 infections, it has been documented that IL-10 production is highly increased in serum. Neutralization of IL-10 activity by treatment with anti-IL-10R antibody rescues T cell responses and consequently virus clearance occurs [2, 31]. Similarly, Cl13-infected IL-10^−/−^ mice show increased T cell function and viral clearance [2, 31]. Thus, IL-10 induces immunosuppression that leads to viral persistence.

IL-10 mechanism of action in viral persistence involves complex cellular cross-talks and interplay between the cytokine source and its target. IL-10^+^ DCs increase in frequency during the acute phase of Cl13 infection and then decline with time [164]. Thus during the acute phase and up to the time that T cell exhaustion is initiated, DCs are the main cellular source of IL-10. Increased IL-10 production by DCs has also been reported during HIV, HCV, and foot-and-mouth disease virus infections, specifically inducing loss of T cell responses [165–170]. Within the DC populations, IL-10 production is higher in CD8*α*^−^ DCs and those expressing high CCR7 levels, a receptor required for DC migration to T cell areas in secondary lymphoid tissues [171]. IL-10 production in these DCs therefore increases the likelihood for IL-10 exerting its regulatory influence on T cells. A similar scenario has been described for HIV in which IL-10-induced immune dysfunction has been related to the modification of DC populations able to gain access to areas where the quality of adaptive immune responses can be profoundly modulated [167]. This mistimed virus-induced IL-10 production by DCs therefore promotes persistent/chronic infections by affecting the inflammatory balance necessary to mount effective T cell responses.

In later stages of chronic Cl13 infection in mice (i.e., from day 8 after infection and throughout the course of disease), NK cells and virus-specific T cells also play a large role in producing IL-10 [72]. In the T cell compartment, virus-specific CD4^+^ T cells become the main IL-10 overproducers. These data are in line with data from other nonviral infections such as* Leishmania* [172], malaria [74], or* Toxoplasma* [75], in which IL-10 produced by T cells has a high impact on disease outcome.

This induction of IL-10 production in CD4^+^ T cells is probably a homeostatic mechanism that limits Th-induced inflammation [173]. IL-10 is induced in Th1 cells obtained from LCMV-nonchronically infected mice after antigen reexposure [72], and chronic antigen exposure can lead to the differentiation of IL-10-producing self-regulatory Th1 cells [90, 91, 174]. Repeated antigen exposure could thus convert virus-specific Th cells into IL-10-producing self-regulatory Th1 cells, a mechanism that could further feed LCMV chronic infections. These self-regulatory Th1 cells can prevent DC maturation and suppress Th1 cell differentiation [102]. This negative feedback mechanism can thus be used by LCMV to suppress Th1 effector function. Similar to self-regulatory Th1 cells, Tr1-like cells have been identified as the main IL-10 producers in HIV infections [175]. Tr1-like cells can also be generated through repeated TCR stimulation in the presence of IL-10 [176], but only when APC are present in the culture [177]. The DC-T cell cross-talk in the presence of high IL-10 levels can thus give rise to IL-10-producing T cells that limit T cell immunity. Hepatitis C virus (HCV) chronically infected patients show an increase in IL-10 production by NK cells [158]. In this case, IL-10-producing NK cells could produce a DC-NK cell cross-talk that impairs adaptive immune response and contributes to chronic infections. It is thus apparent that chronic viral infections often use the regulatory role of IL-10 on T cells and APC to cause T cell exhaustion and deactivate antiviral T cell immunity. Blockade of IL-10R with antibody treatment rescues T cell function and contributes to clearance of persistent infections, suggesting that therapeutic strategies that neutralize IL-10 activity could help control persistent infections, such as HCV, together with other molecular therapies.

### 3.2. Viral IL-10 Homologs in Chronic and Latent Infections

Latency is a mode of persistent or chronic infection in which the viral genome is retained in the host cell, but with a profound restriction on gene expression that results in the production of few viral antigens and no viral particles (reviewed in [178]). Under appropriate conditions, the expression of the viral genome can be induced and infectious particles are produced. To establish latency, viruses have developed immune evasion mechanisms that allow for persistence. Among these mechanisms, large DNA viruses encode for protein homologs of cytokines and chemokines or express viral factors that alter host cytokine production [179, 180]. Members of the representative latency-inducing Herpesviridae family, such as human cytomegalovirus (HCMV) [181], Epstein-Barr virus (EBV) [182], ovine herpesvirus 2 [183], and equine herpesvirus 2 [184], encode for IL-10 homologs. Among the best-characterized IL-10 homologs are the cytomegalovirus-encoded IL-10, termed cmvIL-10, and the latency-associated cmvIL-10, termed LAcmvIL-10 [181, 185] (Figure 1). HCMV is a *β*-herpesvirus that infects a majority of the world's population. Following primary infection, HCMV establishes a lifelong latent infection in cells of the myeloid lineage from where it can later be reactivated to produce infectious progeny [186]. HCMV success in infecting host's cells and causing disease relies partially on a number of virally encoded proteins that are homologs of cellular cytokines, chemokines, and their receptors [181], in which the IL-10 homolog plays an important role. During productive infection cmvIL-10 transcripts are expressed from the gene UL111A [185, 187]. This gene also encodes for the splice variant LAcmvIL-10, which has been associated with latency [188]. cmvIL-10 protein shares 27% amino acid identity with hIL-10 but retains the ability to bind the hIL-10 receptor [187]. Therefore, cmvIL-10 mediates immunomodulatory functions similar to hIL-10 such as inhibiting proinflammatory cytokine production, decreasing MHC-I and MHC-II expression in monocytes [189], and impairing monocyte-derived DCs maturation [190].

Another immunomodulatory mechanism of action for cmvIL-10 resides in its ability to alter macrophage polarization. Depending on the signal they received, monocytes and macrophages become polarized to either M1 proinflammatory or M2 anti-inflammatory subsets [191]. M1 macrophages have a proinflammatory effect with a relevant role in defense against intracellular pathogens. By contrast, M2 macrophages show increased phagocytic activity and suppress proinflammatory cytokine production. cmvIL-10 modulates macrophage polarization and promotes an M2 phenotype [192] characterized by downregulation of MHC-II, upregulation of molecules associated with anti-inflammatory functions, and poor activation of CD4^+^ T cells.

Viral IL-10 homologs probably shape the immune response in the early phase of infection by promoting anti-inflammatory signals. cmvIL-10 induces the upregulation of hIL-10 in monocytes, macrophages, and DCs, thereby amplifying IL-10-mediated immunosuppression and favoring chronicity [193]. Viral Rhesus CMV IL-10 homolog is critical for establishing chronic infections, yet during latent phase a better correlation was observed with cell-derived IL-10 levels than with viral homolog [194]. IL-10-producing CD4^+^ T cells are also linked to HCMV and MCMV persistence [77, 79, 195]. These data indicate that CMV mostly uses endogenous IL-10 signaling to maintain persistence. Taken together these mechanisms enhance the ability of HCMV to establish a primary productive infection and contribute to productive chronic infection.

By contrast, the function of LAcmvIL-10 is much more limited. While both cmvIL-10 and LAcmvIL-10 suppress MHC-II expression on monocytes, LAcmvIL-10 does not impair DC maturation nor does it suppress proinflammatory cytokine production [196, 197]. LAcmvIL-10 can also upregulate hIL-10 in latently infected myeloid cells, although it probably uses a different activation mechanism to cmvIL-10, as LAcmvIL-10 and cmvIL-10 interact differently with the IL-10 receptor and trigger distinct signaling events [196].

Another well-known example of herpesvirus encoding an IL-10 homolog is EBV. EBV is a *γ*-herpesvirus carried by a high percentage of the human population. EBV infections are mostly asymptomatic, but in some cases EBV induces mononucleosis or B cell and epithelial-cell malignancies [198]. One of the strategies used by EBV to establish latent infections is to produce a viral IL-10 (vIL-10) encoded by the BCRF1 gene, classified as a late gene but expressed in B cells early after infection [199]. vIL-10 has been shown to bind to and signal through the human IL-10 receptor, similarly to cmvIL-10 [200], although its affinity for the IL-10 receptor is 1000-fold lower than that of hIL-10 [201]. The lower receptor affinity of vIL-10 compared to hIL-10 does not allow vIL-10 to stimulate the proliferation of thymocytes or mast cells [202, 203], but it retains the capacity to suppress proinflammatory cytokine production and enhance B-cell viability. During EBV infection vIL-10 seems to play a role only during acute infection, during which it protects infected B cells by altering cytokine production, inhibiting CD4 and NK cell responses, and ultimately facilitating EBV dissemination [199, 204].

## 4. Concluding Remarks

IL-10's main function is to prevent immunopathology during inflammatory responses. IL-10 can be produced by virtually all immune cells and in turn IL-10 can modulate the response of these cells. Untangling the complex interactions of this pleiotropic cytokine remains an outstanding challenge for immunologists. IL-10 is so central to immune response regulation that viruses exploit this pathway to evade immunity and establish persistent/latent infections. IL-10 effects in the course of viral infections depend on its spatial and temporal delivery. IL-10 can impair T cell priming in the early stages of adaptive immunity, a mechanism that viruses use to promote their persistence by infecting APC and inducing IL-10 production. The effects of IL-10 on the immune response during acute infections are more subtle. The cytokine is produced in high amounts by antiviral effector T cells at this stage. IL-10 prevents tissue damage in this phase while probably not affecting effector function of antiviral CD8^+^ T cells. IL-10 does however negatively regulate Th1 responses by downmodulating antigen presenting capacity of APC. This regulatory mechanism promotes inflammation resolution when the pathogen clears. Mistiming of IL-10 production at this stage can impair antiviral T cell responses, favoring an early resolution phase that can lead to chronic infection. Chronic antigen exposure in this phase can exhaust antiviral T cells and switch their phenotype to IL-10-producing cells unable to reactivate when presented again with the antigen.

IL-10 blockade, an attractive therapy to treat chronic infection, should be approached with caution since, for instance, IL-10 can be necessary for virus clearance in CNS infection where it controls immunopathology. IL-10 could also play a role in antiviral CD8^+^ T cell memory development; thus IL-10 blockade could prove detrimental to establish long-term CD8^+^ T cell memory. Targeting the fine balance between inflammation and resolution controlled by IL-10 will therefore require spatial and temporal refinement of delivery approaches. A better understanding at the basic level of IL-10 sources and IL-10 effects on the different components of immunity during infections will allow for precise therapeutic targeting of this pathway.
